# The effects of honey solution on postoperative stress, gastric motility, and patient comfort: a randomized controlled trial

**DOI:** 10.3325/cmj.2025.66.204

**Published:** 2025-06

**Authors:** Janja Tarčuković, Lucia Klarica, Mario Dugonjić, Ivan Vuksan, Tomislav Pavlešić, Pavica Šonjić, Lidija Bilić-Zulle, Miroslav Župčić, Alan Šustić, Marko Zelić

**Affiliations:** 1 University of Rijeka, Faculty of Medicine, Department of Anesthesiology, Resuscitation, Emergency and Intensive Care Medicine, Rijeka, Croatia; 2 Clinical Hospital Centre Rijeka, Department of Anesthesiology, Intensive Care and Pain Medicine, Rijeka, Croatia; 3 Clinical Hospital Centre Rijeka, Department of Surgery, Rijeka, Croatia; 4 University of Rijeka, Faculty of Medicine, Department of Surgery, Rijeka, Croatia; 5 University of Rijeka, Faculty of Medicine, Centre for Digestive and Metabolic Medicine, Rijeka, Croatia; 6 University of Rijeka, Faculty of Health Studies, Department of Nursing, Rijeka, Croatia; 7 University of Rijeka, Faculty of Health Studies, Rijeka, Croatia; 8 Croatian Agency for Agriculture and Food, Centre for Viticulture, Enology, and Edible Oils Analysis, Zagreb, Croatia; 9 Clinical Hospital Centre Rijeka, Clinical Department of Laboratory Diagnostics, Rijeka, Croatia; 10 University of Rijeka, Faculty of Medicine, Department of Biomedical Informatics, Rijeka, Croatia; 11 University of Rijeka, Faculty of Health Studies, Department of Clinical Sciences II, Rijeka, Croatia; * Died on October 31, 2023

## Abstract

**Aim:**

To compare the effects of an in-house prepared honey solution (HS) and a commercial carbohydrate beverage (CCB) on postoperative stress response, gastric motility, and self-reported comfort in patients undergoing laparoscopic cholecystectomy.

**Methods:**

This randomized controlled trial enrolled 55 adult patients (the American Society of Anesthesiologists physical status I-II) undergoing laparoscopic cholecystectomy at the University Hospital Center Rijeka from January 1, 2022, to December 31, 2022. Participants were randomly assigned to receive either HS (n = 20), prepared from diluted chestnut honey, or CCB (n = 35). Both solutions were administered in the volumes of 800 mL the evening before surgery and 400 mL two hours before anesthesia. Stress response and inflammation were assessed by measuring cortisol and interleukin 6 levels at six standardized time points. Gastric motility was evaluated with a paracetamol absorption test, and patient-reported outcomes were recorded postoperatively using a visual analogue scale.

**Results:**

The HS group exhibited higher cortisol and interleukin 6 levels at multiple perioperative time points compared with the CCB group (*P* < 0.05). However, they had significantly improved gastric motility, with higher paracetamol concentrations 15 to 180 minutes post-ingestion (*P* = 0.028-0.001). Additionally, HS group reported reduced thirst, nausea, and pain, but had lower appetite and well-being scores.

**Conclusion:**

While HS represents a potential natural and cost-effective alternative to CCB, further studies are needed to determine its role within preoperative nutrition strategies for enhanced recovery after surgery.

**Trial identification number:**

ISRCTN11350865.

Preoperative fasting is a fundamental principle of surgical preparation, with patients traditionally being required to abstain from eating and drinking before surgery to reduce the risk of pulmonary aspiration. Historically, 12-16 hours of fasting has been the standard practice, primarily based on concerns regarding residual gastric contents (1-3). However, while effective in minimizing aspiration risk, prolonged fasting imposes considerable physiological and psychological burden on patients, contributing to stress response, dehydration, and postoperative insulin resistance (4-6).

Advances in the understanding of perioperative (patho)physiology, alongside safer airway management techniques, have led to a paradigm shift toward more liberal preoperative fasting guidelines, which emphasize the benefits of preoperative carbohydrate loading (6,7). According to current guidelines, clear carbohydrate-containing fluids can be safely consumed up to two hours before elective surgery, supported by evidence demonstrating their role in improving metabolic stability, reducing postoperative nausea and vomiting, and enhancing patient comfort (7-9). Additionally, clear liquids generally empty from the stomach of healthy individuals within 90 minutes, after which the risk of aspiration decreases. However, individual adjustment is needed for patients with diabetes, obesity, or other conditions that might slow gastric emptying (10).

Among various commercially available clear carbohydrate beverages, honey represents a natural alternative that could offer several potential advantages in preoperative nutrition. Honey has long been used for both nutritional and therapeutic purposes. It mainly consists of glucose and fructose, with smaller amounts of sucrose and more than 200 bioactive compounds, including minerals, organic acids (formic, oxalic, lactic, and citric acids), water, and enzymes such as invertase, diastase, and amylase (11-15). Its antioxidant, antimicrobial, and anti-inflammatory properties make it suitable for therapeutic applications (16-28).

As honey might affect metabolism and digestive processes, it is a viable candidate for preoperative carbohydrate loading in abdominal surgery. However, evidence remains limited. To our knowledge, only two trials have evaluated honey-based solutions in this context (29,30). One study in open colorectal surgery demonstrated reduced insulin resistance and perioperative discomfort in patients who consumed a honey drink compared with overnight fasting (29). Another recent trial in elective intraabdominal surgery reported comparable glycemic control, gastric emptying, and recovery parameters to standard maltodextrin-based loading (29,30). However, both studies involved heterogeneous procedures and limited outcome assessment. This study is the first to evaluate the chestnut honey as a standardized, natural carbohydrate-loading agent in elective laparoscopic surgery, using a formulation matched in carbohydrate content to a commercial preparation and incorporating comprehensive physiological and patient-centered outcome measures.

## Participants and methods

### Participants

This randomized controlled study enrolled adult patients (18 years or older) with ASA physical status I-II who were scheduled for elective laparoscopic cholecystectomy. The study was conducted at the Department of Surgery, University Hospital Centre Rijeka, Croatia, from January 1, 2022 to December 31, 2022. Participants received either honey solution (HS), prepared from diluted chestnut honey, or commercial carbohydrate beverage (CCB).

The exclusion criteria were any history of neurosurgical procedures or head trauma, previous hepatobiliary or gastrointestinal surgery, chronic kidney disease (creatinine clearance <50 mL/min), hepatobiliary disease (eg, cholecystitis, pancreatitis) within the last 6 months, and acute hepatobiliary issues with severe laboratory deviations, known drug allergies, or adverse reactions related to the studied medications, diabetes, severe chronic obstructive pulmonary disease, significant cardiovascular disease (myocardial infarction within 3 weeks, preoperative left ventricular ejection fraction <40%), advanced malignant disease, and surgical emergencies. Additionally, patients were excluded if intraoperative findings revealed gallbladder inflammation or if a conversion to open cholecystectomy was required.

The study was approved by the Ethics Committee of the Clinical Hospital Center Rijeka. The trial is registered in the ISRCTN registry under the identifier ISRCTN11350865. Written consent was obtained from all participants or their legal representatives.

### Honey analysis

The type of honey used in the study was the Lovran maroon honey (*Castanea sativa Mill*.), collected from the slopes of Učka, in Liganj, Primorsko-Goranska County (45°18′53˝N 14°17′02˝E). This variety is dark red in color and tastes distinctly bitter-sweet. Laboratory analysis was performed in accordance with the International Honey Commission (IHC) guidelines (31). The physicochemical properties of the honey (electrical conductivity, water content, pH, free acids, hydroxymethylfurfural, reducing sugars, sucrose, and pollen composition) are presented in the supplemental material.

The electrical conductivity of honey was determined by dissolving 20 g of honey in 100 mL of distilled water at 20 °C, followed by measurement with an electrical conductivity cell, as per established protocol. Water content was analyzed by refractometry per an established protocol by using a digital refractometer PAL22S (Atago, Tokyo, Japan), after dissolving honey in a heating bath at 50 °C and allowing it to equilibrate at 20 °C for six minutes. The pH value was measured with a pH meter (Mettler Toledo, Columbus, OH, USA), while free acidity was determined through titration with 0.1 M sodium hydroxide until reaching a pH of 8.3, as per the official IHC method. The hydroxymethylfurfural (HMF) content was assessed by using high-performance liquid chromatography (HPLC/DAD, Agilent 1200, Santa Clara, CA, USA), following the standardized method for HMF quantification by HPLC. A water-methanol mobile phase (9:1, V/V) with a flow rate of 1.0 mL/min was used, and sample content was determined by comparing peak areas with standard reference solutions. The contents of reducing sugar and sucrose were quantified by using Fehling’s reagent titration, while sucrose concentration was determined by calculating the difference in total reducing sugars before and after acid hydrolysis (31). The pollen composition was assessed microscopically after dissolving 10 g of honey in 20 mL of distilled water, followed by centrifugation at 3500 rpm for 15 minutes and sediment collection for microscopic analysis (32,33). All sample tests were run in duplicate.

To ensure nutritional equivalence with the CCB, HS was formulated to match its carbohydrate content and approximate caloric load. On the evening before surgery, patients received a dose containing 100 g of carbohydrates, achieved by dissolving 130 g of honey in 800 mL in warm (20-25 °C) sterile water, providing approximately 400 calories. A second preoperative dose, administered two hours before anesthesia, consisted of 65 g of honey dissolved in 400 mL of sterile water, delivering 50 g of carbohydrates and approximately 200 kcal. This mirrored the CCB protocol, in which patients received 800 mL containing 100 g of carbohydrates (400 kcal) the evening before surgery and 400 mL containing 50 g of carbohydrates (200 kcal) two hours preoperatively.

### Perioperative assessment

Upon hospital admission, baseline demographic and clinical data were recorded for all participants. The participants were then randomly assigned to receive either an in-house prepared HS or CCB. Randomization was conducted by one of the investigators who was not in direct contact with the patients or their outcome assessment, with a computer-generated random sequence. The ingredients of the commercial carbohydrate beverage, Nutricia preOp® (Nutricia Advanced Medical Nutrition, Zoetermeer, The Netherlands) are listed in the supplemental material. Both solutions were administered in two doses – 800 mL the evening before surgery and 400 mL two hours before anesthesia induction. No solid foods were allowed after midnight, following standard hospital fasting protocols.

Intraoperatively, the procedures were performed according to the standard laparoscopic cholecystectomy protocol. A balanced general anesthetic technique was used in all cases. A nasogastric tube was inserted after anesthesia induction and before insufflation of CO_2_ to ensure minimal residual gastric volume.

Metabolic and stress parameters were measured to assess the physiological response associated with each preoperative solution. Blood samples were collected by venipuncture in test tubes with clot activator and gel separator (Greiner Bio-One GmbH, Kremsmünster, Austria), then centrifuged at 3500 rpm for 10 minutes. Serum samples were used for all analyses. Cortisol levels were measured twice daily (08:00 and 16:00) on the day before surgery (T1 and T2, respectively), on the day of surgery (T3 and T4, respectively), and on the first postoperative day (T5 and T6, respectively) to account for normal circadian variations. Interleukin 6 (IL-6) levels were determined at four time points: on the day before surgery (T1), in the morning (T2) and afternoon (T3) on the day of surgery, and in the morning (T4) of the first postoperative day. Both parameters were measured with electrochemiluminescence immunoassays: IL-6 with Roche Cobas e411 and cortisol with Cobas e601 (Roche Diagnostics GmbH, Mannheim, Germany).

To assess gastric motility, patients underwent a paracetamol absorption test at 16:00 on the day of surgery. Each patient consumed 1 g of Paracetamol JGL® syrup (120 mg/5 mL; Jadran-Galenski Laboratorij, Rijeka, Croatia), after which blood samples were drawn at baseline and at 15, 30, 60, 120, and 180 minutes post-ingestion (T0’, T15’, T30’, T60’, T120’, T180’, respectively). Paracetamol concentrations were assessed simultaneously for all samples using the ACET2 assay on a COBAS e501 analyzer (Roche Diagnostics GmbH).

On the first postoperative day, patient-reported outcomes were assessed using a visual analogue scale (VAS). Thirst, nausea, vomiting, and pain were rated on a 0-10 scale, with 0 indicating no symptoms and 10 representing the most severe intensity. Appetite and general well-being were rated with 10 as the best possible state and 0 as the lowest. All scores were recorded and compared between the groups. The timeline of preoperative carbohydrate administration, sample collection, and assessment of patients-reported outcomes is shown in Supplemental Table 2.

### Statistical analysis

Data are presented as means with standard deviations or medians with ranges. Data distribution was assessed with a Kolmogorov-Smirnov test. Differences between the groups in continuous variables were assessed with independent and paired *t* tests or Mann-Whitney U tests. Wilcoxon tests were applied to paired dependent samples, while differences in categorical variables were assessed with χ^2^ tests with Yates’ correction or Fisher exact test. A *P* value of <0.05 was considered statistically significant. All statistical analyses were performed with GraphPad Prism (version 10.4.0, GraphPad Software, San Diego, CA, USA).

## Results

The final sample included 55 patients: 35 were assigned to the CCB group and 20 to the HS group. No intraoperative exclusions and no adverse events related to aspiration or regurgitation were reported. Aspiration volumes did not significantly differ between the groups.

The mean age was 50 years in the HS group and 48 years in the CCB group. The HS group included 25% male patients, while the CCB group included 33% male patients. There were no baseline significant differences between the groups in terms of age, sex distribution, or any of the measured parameters, except for IL-6 levels.

**Figure 1:**
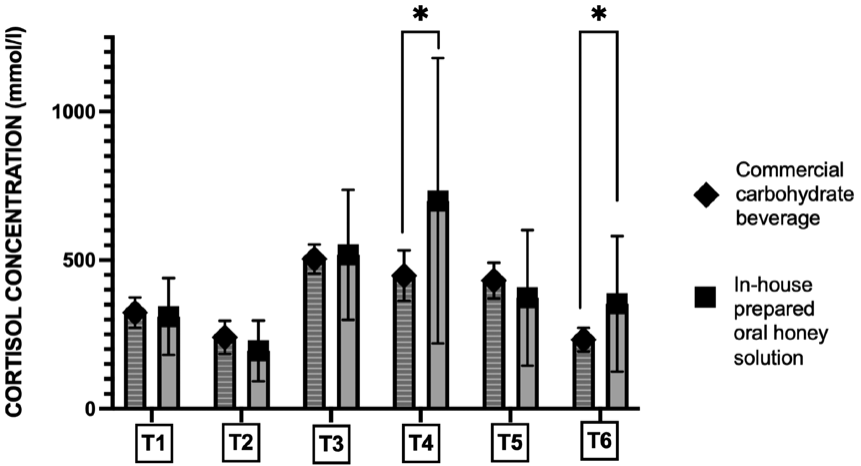
Cortisol concentrations in patients receiving commercial carbohydrate beverage and those receiving honey solution. Measurements were made at six time points: T1 (day before surgery, 08:00), T2 (day before surgery, 16:00), T3 (day of surgery, 08:00), T4 (day of surgery, 16:00), T5 (first postoperative day, 08:00), and T6 (first postoperative day, 16:00). Data are expressed as means ± standard deviations. Asterisks indicate significant differences.

To assess the stress response, cortisol concentrations were measured at six predefined time points (T1-T6) (Figure 1). The HS group exhibited significantly higher cortisol levels at T4 (448 ± 85 vs 699.7 ± 480 μg/L; *P* = 0.031) and T6 (232 ± 40 vs 352.7 ± 200.4 μg/L; *P* = 0.031). (Table 1).

**Table 1:** Cortisol concentrations in patients who received commercial carbohydrate beverage and those who received honey solution

Time point		Cortisol concentration (μg/L) in		
		commercial carbohydrate beverage group	in-house prepared oral honey solution group	P
Day before surgery	8:00	323 ± 51	310 ± 129.2	0.670
	16:00	240 ± 56	194.6 ± 102.3	0.078
Day of surgery	8:00	504 ± 49	517.5 ± 218.7	0.788
	16:00	448 ± 85	699.7 ± 480	0.031
First postoperative day	8:00	431 ± 60	372.9 ± 228.2	0.277
	16:00	232 ± 40	352.7 ± 200.4	0.031

**Figure 2:**
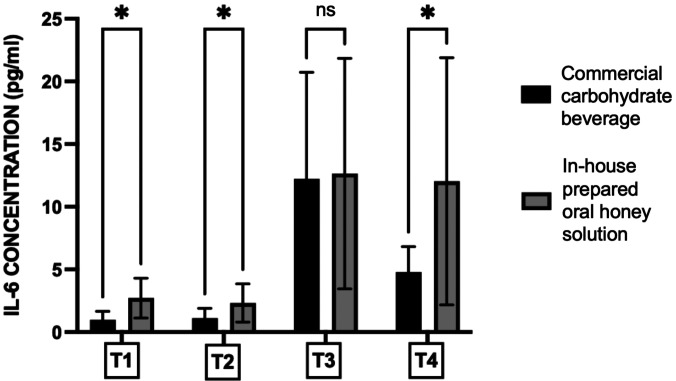
Interleukin 6 concentrations in patients receiving commercial carbohydrate beverage and those receiving honey solution. Measurements were made at four time points: T1 (day before surgery, 08:00), T2 (day of surgery, 08:00), T3 (day of surgery, 16:00), and T4 (first postoperative day, 08:00). Data are expressed as means ± standard deviations. Asterisks indicate significant differences.

To evaluate the inflammatory component of stress response, IL-6 levels were measured at four predefined time points (T1-T4) (Figure 2). The HS group had significantly higher IL-6 levels at T1 (0.98 ± 0.68 vs 2.72 ± 1.59 pg/mL; *P* < 0.001), T2 (1.1 ± 0.8 vs 2.32 ± 1.53 pg/mL; *P* = 0.003), and T4 (4.79 ± 2.03 vs 12.03 ± 9.86 pg/mL; *P* = 0.004) (Table 2).

**Table 2:** Interleukin 6 concentrations in patients who received commercial carbohydrate beverage and those who received honey solution

Time point		IL-6 levels (pg/mL)		
		commercial carbohydrate beverage group	in-house prepared oral honey solution group	P
Day before surgery	8:00	0.98 ± 0.68	2.72 ± 1.59	<0.001
Day of surgery	8:00	1.1 ± 0.8	2.32 ± 1.53	0.003
	16:00	12.22 ± 8.51	12.64 ± 9.2	0.868
First postoperative day	8:00	4.79 ± 2.03	12.03 ± 9.86	0.004

**Figure 3:**
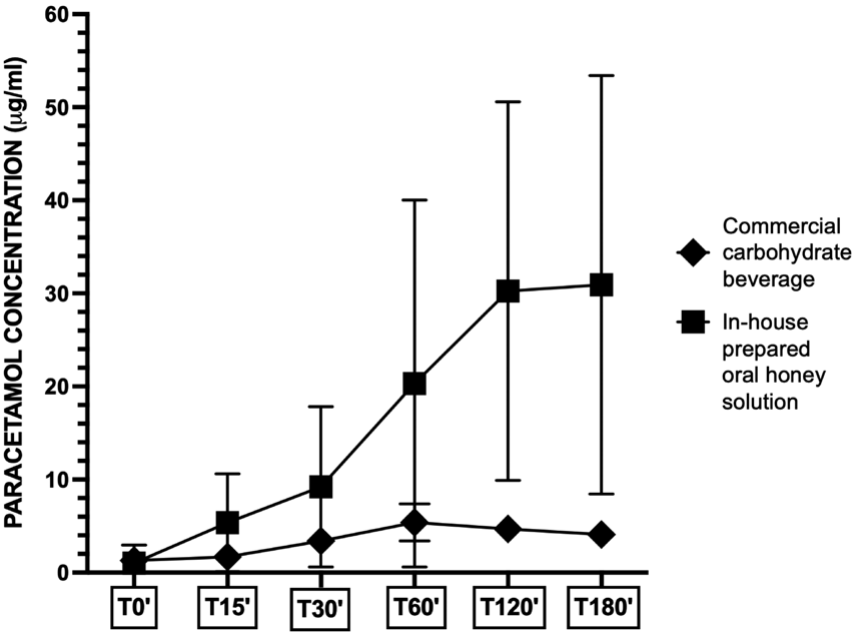
Paracetamol absorption test results in patients receiving commercial carbohydrate beverage and those receiving honey solution. Paracetamol syrup (1 g) was administered at 16:00 on the day of surgery, and blood samples were collected at six time points: T0’ (baseline, immediately before ingestion), T15’ (15 minutes after ingestion), T30’ (30 minutes), T60’ (60 minutes), T120’ (120 minutes), and T180’ (180 minutes). Data are expressed as means ± standard deviations.

Gastric motility was indirectly assessed after surgery with the paracetamol absorption test. Measurements at baseline (T0’) and 15, 30, 60, 120, and 180 minutes (T15’-T180’) post-ingestion are presented in Figure 3. Baseline paracetamol levels did not differ significantly between the CCB and HS group (1.3 ± 0.2 vs 1.0 ± 2.0 mg/L; *P* = 0.615). However, from T15’ onward, the HS group showed significantly higher paracetamol concentrations at T15’ (*P* = 0.028); T30’ (*P* = 0.032); T60’ (*P* = 0.018); T120’ (*P* = 0.001); and T180’ (*P* = 0.001). These findings suggest faster absorption, indicative of improved gastric emptying, in patients who received HS.

**Figure 4:**
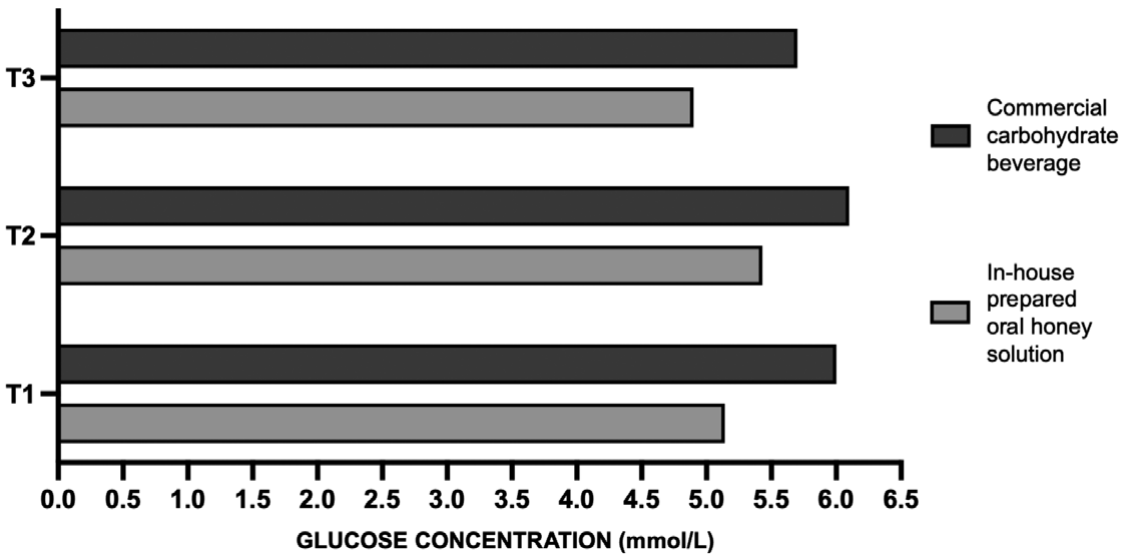
Glucose concentrations in patients receiving commercial carbohydrate beverage and those receiving honey solution, measured at three time points: T1 (day before surgery), T2 (day of surgery), and T3 (day after surgery). Each point represents the mean glucose level for the respective group.

Glucose levels were measured at three time points (T1, T2, T3). Patients who received HS had slightly lower values than the CCB group at all time points; however, the difference was not significant (T1: 5.14 mmol/L vs 6.0 mmol/L; T2: 5.43 vs 6.1 mmol/L; T3: 4.9 vs 5.7 mmol/L) (Figure 4).

**Figure 5:**
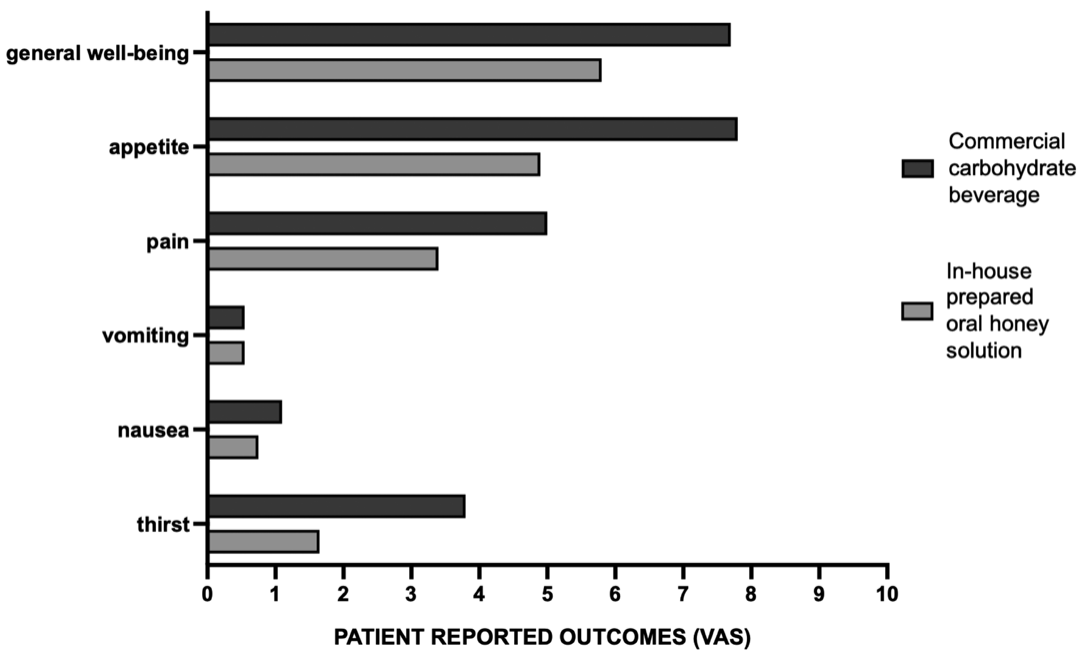
Patient-reported outcomes on the first postoperative day in patients receiving commercial carbohydrate beverage and those receiving honey solution. Thirst, nausea, vomiting, and pain were scored on a visual analog scale (VAS) from 0 (no symptom) to 10 (worst possible), whereas appetite and general well-being were measured on a reversed scale from 0 (lowest) to 10 (highest). Each column shows the mean score for the respective group.

Compared with the CCB, the HS group reported lower VAS scores for thirst (mean 1.65 vs 3.8), nausea (0.75 vs 1.1), and pain (3.4 vs 5.0). Vomiting scores were similar in both groups (0.55 vs 0.55). However, patients in the CCB group reported higher appetite score (7.8 vs 4.9) and general well-being score (7.7 vs 5.8) (Figure 5).

## Discussion

While our findings indicate better gastric emptying and improved symptom relief in the HS group, they also suggest higher postoperative stress and inflammatory markers, raising questions about the metabolic effects of honey during surgical recovery.

Postoperative cortisol and IL-6 levels were higher in the HS group. Cortisol is a well-known marker of physiological stress, and elevated levels postoperatively suggest a stronger stress response to surgical trauma (34,35). Under physiological conditions, cortisol follows a circadian rhythm, peaking in the morning and decreasing throughout the day. However, in the HS group, cortisol did not follow this pattern. In particular, cortisol levels were not only significantly higher in the afternoon on the day of surgery and the following day, but the expected decline on the day of surgery was also absent. Interestingly, despite overall higher levels, cortisol in the HS group was lower on the morning after surgery than in the CCB group. These fluctuations suggest a complex interaction with metabolic pathways. While honey may initially heighten stress, later it can affect cortisol regulation differently.

IL-6, a pro-inflammatory cytokine, plays a key role in surgical stress and peaks after major procedures (36,37). IL-6 levels were significantly higher in the HS group at baseline (T1) and remained elevated at T2 and T4, suggesting a preexisting difference in inflammatory status. This baseline imbalance limits the interpretability of postoperative IL-6 trends, as elevated values in the HS group may reflect both the intervention and the underlying variability, which should be acknowledged as a limitation of the study. Although the largest difference in IL-6 occurred on the first postoperative day, it cannot be conclusively attributed to the type of carbohydrate loading alone. Therefore, further studies should account for baseline cytokine levels to more accurately assess the specific impact of nutritional interventions on postoperative inflammation.

Honey contains polyphenols, coumarins, and enzymes that might modulate immunity, but their role in perioperative inflammation remains unclear (38-40). In contrast, while synthetic, CCB may provide a more controlled metabolic response, which potentially explains the lower stress and inflammatory markers in that group. However, it remains unclear whether these transient elevations in markers translate to meaningful clinical consequences. Future studies should explore whether honey's carbohydrate makeup, especially its high fructose content, affects metabolic demands that influence surgical recovery.

In contrast to stress markers, honey significantly improved gastric motility compared with CCB. Patients in the HS group had higher serum paracetamol concentrations at all time points, which indicates faster gastric emptying. Studies confirm that preoperative carbohydrate beverages do not delay gastric clearance (41-43). However, it is particularly interesting that honey surpassed CCB in this regard, possibly due to its glucose-fructose composition, which may promote quicker passage through the stomach compared with CCB’s maltodextrin content (44,45). Additionally, honey’s ability to stimulate digestive enzyme activity could further accelerate transit. This could explain why gastric emptying was faster in the honey group (46). Experimental studies also suggest that honey has gastroprotective properties, which could further enhance motility (47). While these findings suggest honey could be an effective carbohydrate loading option, more research is needed to show how this impacts broader surgical outcomes.

Patient-reported outcomes also followed a clear trend. Patients in the HS group reported reduced thirst, nausea, and pain scores on the first postoperative day, which supports the theory that preoperative intake of honey-based carbohydrate solution helps counteract some discomfort after surgery (9,41,48). However, the HS group also reported lower appetite and well-being scores, which is a less expected finding. While the exact reason remains unclear, the finding may be explained by the effect of honey-induced metabolic changes, including cortisol fluctuations, on hunger regulation and overall energy balance. Perioperative carbohydrate loading is reported to impact satiety hormones, but direct comparisons between honey and synthetic carbohydrate solutions are lacking (9,49). Taken together, while honey appears to improve postoperative comfort, its effects on appetite and stress responses warrant further research.

This study has several limitations. First, the single-center design may limit the generalizability of the findings. Second, the sample size is relatively small, as it was limited by strict inclusion criteria and logistical challenges in coordinating standardized preoperative feeding, surgery timing, and perioperative assessment within a single-center setting. A larger, multicenter trial would help determine whether honey’s effects on gastric emptying and stress response hold in larger populations. Additionally, we measured short-term effects only, and long-term recovery, wound healing, infection rates, and hospital stay durations remain unknown. Future studies should include extended follow-up to assess honey’s broader surgical impact. Honey composition also varies between sources, making standardization a concern. Although our sample was characterized under strict laboratory conditions, different honey types may produce varying effects. Future research should explore the role of standardized honey formulations for medical use.

This study provides new insights into honey as a potential alternative for preoperative carbohydrate loading, showing marked benefits in gastric motility and symptom relief. However, the elevated cortisol and IL-6 levels in the honey group suggest it may not suppress postoperative stress and inflammation as efficiently as synthetic carbohydrate solutions.

Given honey’s natural composition, accessibility, and potential cost-effectiveness, its role in preoperative nutrition remains promising. However, its impact on metabolism, stress response, and long-term outcomes demands further research. Future studies should focus on larger patient cohorts, extended postoperative monitoring, and metabolic profiling to fully assess honey’s place in perioperative care and enhanced recovery protocols.
